# Massive malignant solitary fibrous tumor of the diaphragm: A case report

**DOI:** 10.1097/MD.0000000000018992

**Published:** 2020-01-31

**Authors:** Dan Liu, Yun Wang, Yu Zheng, Han-Lu Zhang, Zi-Hao Wang

**Affiliations:** a Division of Thoracic Surgery, West China Hospital of Sichuan University, Chengdu City; b Department of The First Surgery, The Traditional Chinese Medical Hospital, AnYue City, Sichuan Province, PR China.

**Keywords:** diaphragmatic reconstruction, immunohistochemistry, malignant solitary fibrous tumor, primary diaphragmatic tumor, thoracotomy

## Abstract

**Introduction:**

Malignant solitary fibrous tumor (MSFT) of the diaphragm is extremely rare, and to the best of our knowledge, only three cases have been reported in the past two decades. In all these cases, the diaphragms were usually reconstructed with artificial diaphragm patch because of the extensive resection.

**Patient concerns:**

We reported a male patient with complaints of dyspnea, chest pain and massive pleural effusion in the left chest detected by chest X-ray. A huge mass of 20 × 20 cm was seen in the left lower chest in the computed tomography (CT) scan.

**Diagnosis:**

The diagnosis of MSFT originating in the diaphragm was made by post-operative immunohistochemical examination.

**Interventions:**

After draining 4000 ml of pleural effusion by Pleurx catheter to relieve the pressure symptom, the patient underwent en-block resection by left posterolateral thoracotomy. A pedicle tumor originating in the left diaphragm was found, which was smooth, lobular, did not invade surrounding tissues or organs, and received blood supply from the left phrenic vessels. The diaphragm was successfully sutured without tension and did not require artificial reconstruction as the defect was small.

**Outcomes:**

After 2 months follow-up, the left lung was restored to normalcy, and no pleural effusion or new occupying neoplasm was found in follow-up CT.

## 1 Introduction

Primary diaphragmatic tumor and MSFT are rare diseases, while MSFT originating in the diaphragm is extremely rare.^[1–3]^ Due to the extremely low incidence of these diseases, very few cases have been reported till date, with only three cases of MSFT originating in the diaphragm reported in PUBMED during the past two decades.^[4–6]^ There is insufficient data for statistical analysis, which also hinders complete understanding of the pathogenic factors, clinical characteristics, imaging characteristics, pathological characteristics, treatment methods and prognostic analysis. We recently treated a patient with MSFT originating in the left diaphragm, who had massive pleural effusion. The tumor was pedicle and successfully en-block resected, the diaphragm was successfully reconstructed without artificial material because it could be sutured without tension. Unlike other huge MSFTs that generally originate in pleural and have a broad base, our case was a pedicle tumor that originated in the left diaphragm. This case indicated that the possibility of MSFT should be considered for giant tumors originating in the diaphragm, and immunohistochemical examination is required for diagnosis. It is possible to easily and completely remove massive MSFTs of the diaphragm without diaphragm reconstruction with artificial material if they are pedicled. This report may provide a reference resource for the diagnosis and treatment of this disease.

## 2 Case presentation

**Figure 1:**
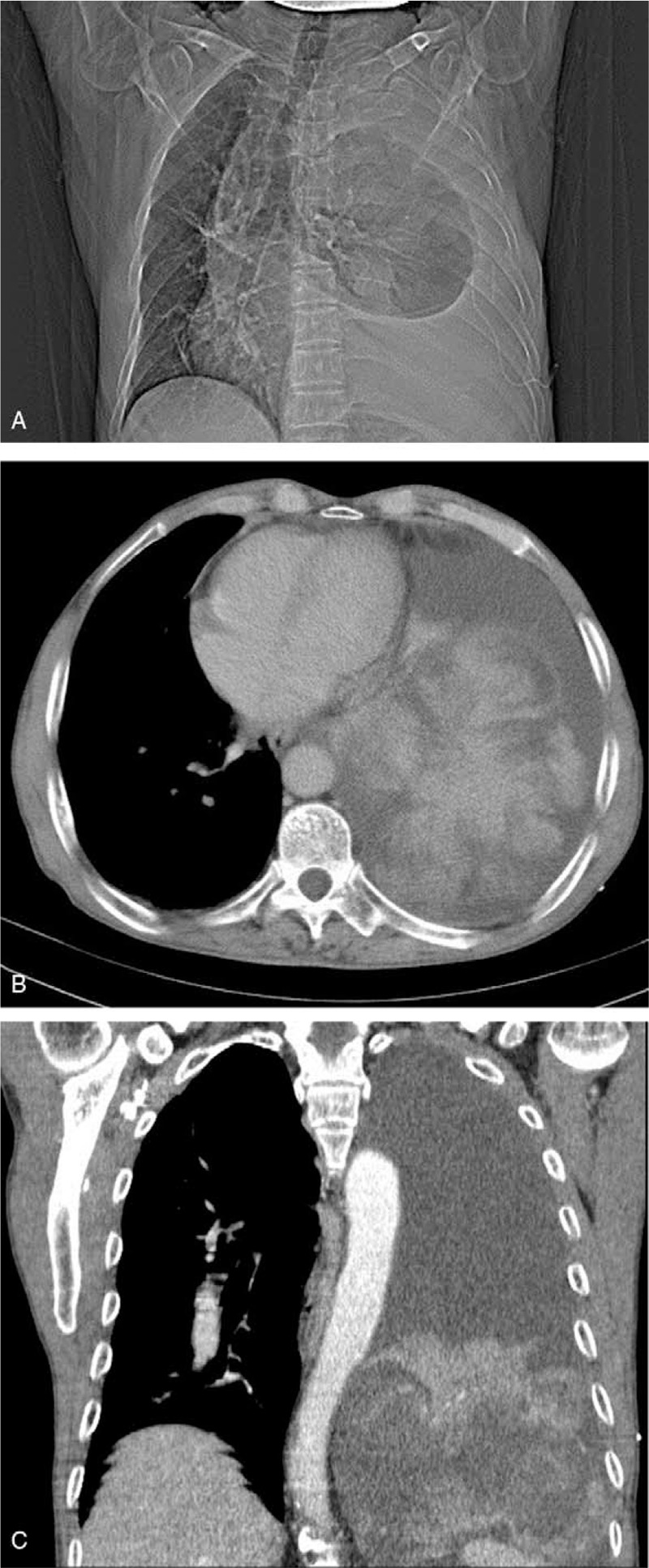
A1. X-ray showed that the pleural effusion seriously compressed the left lung and the mediastinum organs, the aorta and the trachea were extremely shifted to the right side. A2-A3. CT scan showed clear border, irregular lobular shadow and hemorrhagic-necrotic foci after enhancement, along with twisted vascular shadow.

**Figure 2:**
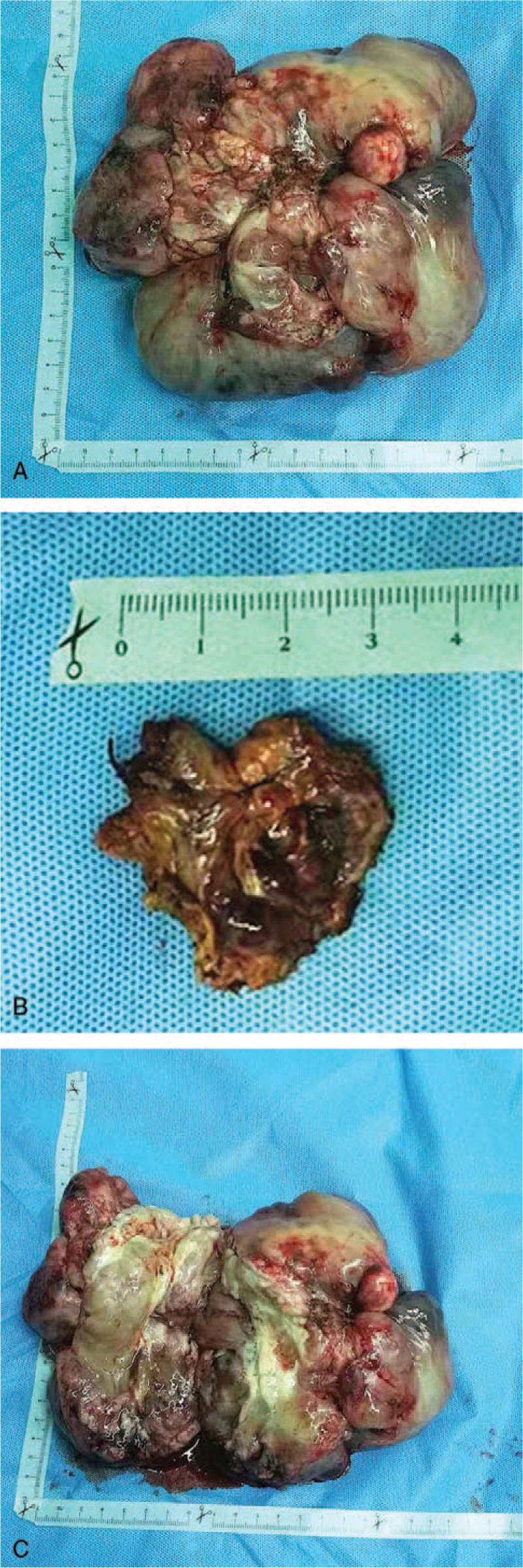
B1. Showed the tumor with the size of 20 × 20 cm. B2. Showed the base of the diaphragm of 2 × 2 cm. B3. Showed the hemorrhagic-necrotic lesions of the tumor section.

**Figure 3:**
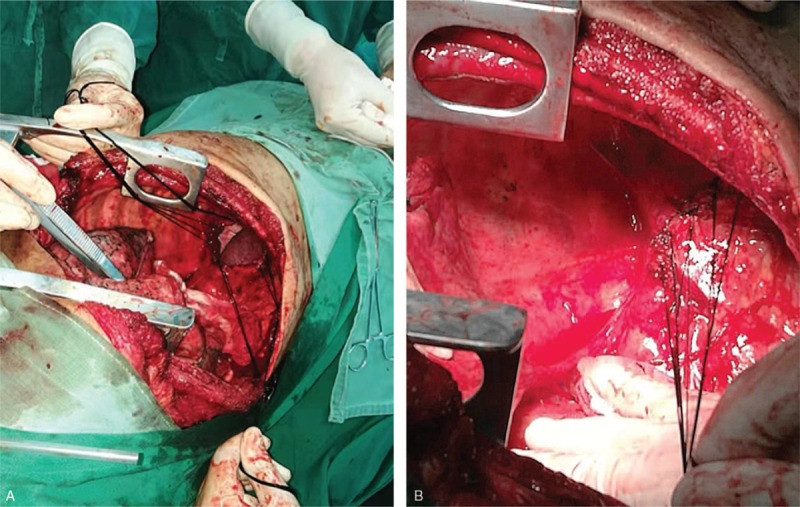
C1. Showed the defect of the diaphragm. C2. Showed the reconstruction of the diaphragm without artificial materials.

**Figure 4:**
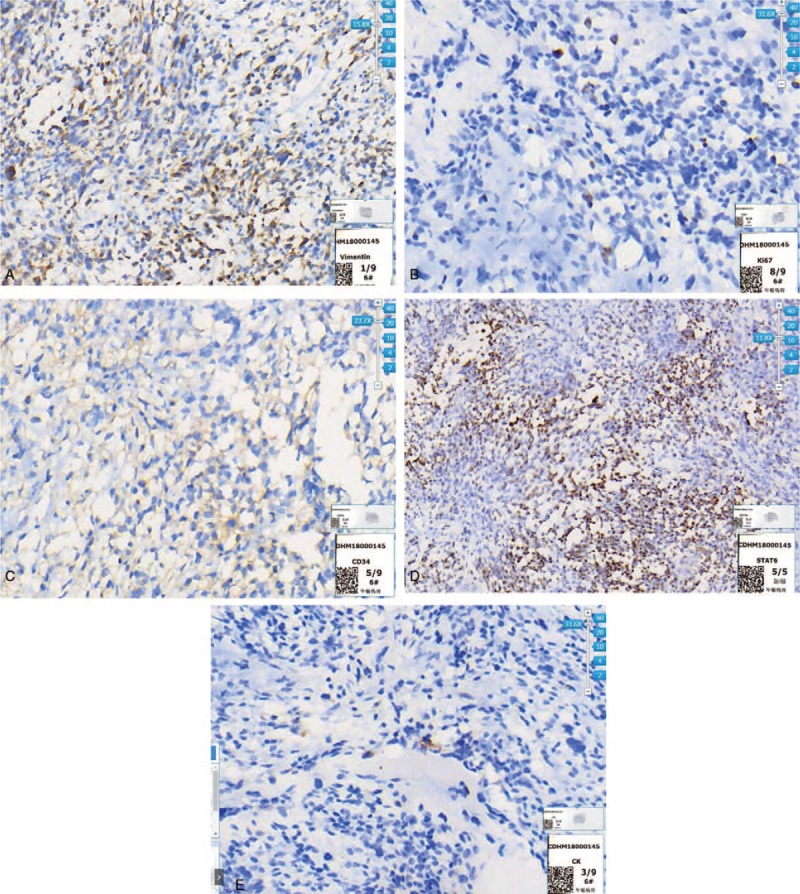
D1. Few short spindle cells were seen under the microscope, and few large cells and multinucleated giant cells with deep nuclear staining. Obvious bleeding and necrosis was seen between tissues. Showed the immunohistochemical staining showed Vimentin (+). D2. The immunohistochemical staining showed Ki67 (+, 10%). D3. The immunohistochemical staining showed CD34 (focal+). D4. The immunohistochemical staining showed STAT (focal+). D5. The immunohistochemical staining showed, CR (focal+).

A 73-year-old male with no history of alcohol and tobacco use complained of dyspnea, chest and epigastric pain, accompanied by reduced mobility for two weeks. The X-ray film showed massive pleural effusion in the left chest, which seriously compressed the left lung, and the mediastinum was extremely shifted to the right side. Contrast-enhanced CT scan showed a mass of 20 × 20 cm, bulging into the left lower thoracic cavity, with clear border separating the tumor from the surrounding tissue. Moderate enhancement and twisted vascular shadow with “pseudocapsule” signs were seen (Fig. 1 A1–A3). T-cell subgroup count: CD4 268/ul↓, CD8 216/ul↓, CD3 544/ul↓, tumor markers: carbohydrate antigen (CA125) 525.6U/ml↑, CA72 419.90U/ml↑, and circulating tumor cells (CTC): 8.7 Fu/3 ml. PleurX-catheter was inserted into the left chest cavity to relieve the symptoms of oppression after admission. The drainage fluid was light red, clear and no tumor cells were found. After continuous drainage of 4000 ml from the left chest, the symptom of dyspnea was improved and video-assisted thoracoscopic surgery (VATS) was subsequently performed. There was no neoplasm in visceral and parietal pleura, and the tumor was too large to observe and difficult to treat by VATS. Traditional thoracotomy was performed in the fifth-sixth intercostal posterolateral incision. The mass was supplied by the left phrenic blood vessels, and had obvious pedicle derived from the left diaphragm. The mass was 20 × 20 cm in size, and weighed 1500 g. The surface of the mass was smooth and lobular, the section of the tumor was grayish white, and a pedicle of 2 × 2 cm was located in the central part of the left diaphragm (Fig. 2 B1–B3). The intrathoracic adhesion was excised, the branch of the diaphragm blood vessel that supplied the tumor was ligated, and the tumor was completely removed from the diaphragm. At the base of the tumor, which originated from the diaphragm, a 2 cm section at the margin was excised and no tumor cells were found in the incised margin by intraoperative frozen section. The defect of diaphragm was about 5 × 5 cm, which could be sutured without tension. Hence, the diaphragm was directly sutured for reconstruction, without artificial materials (Fig. 3 C1-C2). The patient recovered smoothly after the operation, the pleural effusion disappeared, the lung was restored, and the incision healed in one stage. Pathological examination showed few short spindle cells under the microscope, and a few large cells and multinucleated giant cells with deep nuclear staining, along with obvious bleeding and necrotic lesion between tissues. Immunohistochemical examination showed Vimentin (positive), CD34 (focal positive), STAT (focal positive), CR (focal positive) and Ki67 (10% positive). All these signs were consistent with MSFT, and surgical margin was not invaded by the tumor (Fig. 4 D1–D5). After 2 months follow-up, the left lung was restored, and no pleural effusion or new occupying neoplasm was found in CT.

## 3 Discussion

Primary diaphragm tumors are rare, including sarcoma, fibrosarcoma, schwannoma, leiomyosarcoma, germ cell tumor, etc.^[2,7–12]^ MSFT is also a rare disease that was previously thought to originate from mesothelial tissue, especially pleura and peritoneum, but has been subsequently agreed to originate from mesenchymal tissue and dendritic mesenchymal cells in any part of the body including lung, liver, central nervous system and other organs.^[13–15]^ There are only three cases reported in PUBMED of MSFT originating from the diaphragm in the past 20 years. Hence, there is insufficient data for statistical analysis, which hinders complete understanding of the pathogenic factors, clinical characteristics, imaging characteristics, pathological characteristics, treatment methods and prognostic analysis.

MSFT is often asymptomatic, occurs more commonly in middle-aged patients, aged 50 to 75 years, with no gender differences, and grows slowly in the form of a lump. As the tumor grows, the patients show symptoms such as cough, pain, dyspnea, corresponding pulmonary osteoarthropathy, and a few cases develop tumor syndrome, such as paraneoplastic syndrome and hypoglycemia caused by insulin-like growth factor (ILGF), etc.^[1,6,13]^ In addition to the typical symptoms, our patient had massive pleural effusion in the left side. Cytological examination of pleural effusion and CT-guided puncture biopsy found nothing unusual except inflammatory tissues. More cases and studies are needed in the future to verify whether inflammatory tissues are associated with mass necrosis of the tumor.

MSFT generally manifests as a huge mass bulging into the chest in CT, and is linked to the pleural with wide base. The tumor is characterized by diameter >10 cm, isolation, clear and smooth margin, lobulated shallow, uniform density or necrosis; and contrast-enhanced CT shows moderate enhancement, twisted vascular shadow and “pseudocapsule” sign.^[16–17]^ The high density area is caused by the rich vascular network and the low density area is caused by the necrotic components and sparse area of cells, the presence of the pedicle tumor is rarely identified on CT but can be inferred by evaluating lesion mobility.^[18]^ Our patient's contrast-enhanced CT scan showed the mass was over 20 × 20 cm diameter, with clear border, moderate enhancement, “pseudocapsule” signs, all these characteristics was in line with the character MSFT. Hasegawad and colleague suggested that the diagnosis of extrapleural malignant solitary fibroma depended more on microscopic morphological characteristics: abundant cells and increased density, the morphology of the cells is diverse and the heteromorphism is obvious, mitotic images are easy to see (4∼10HPF).^[19]^ Sun and colleague reported that CD34 and Ki67 was an important diagnostic basis, especially the level of Ki67 was positively correlated with the malignant degree of the tumor, the average value of benign solitary fibroma was 1.9%, while that of malignant Ki67 was 6.11%.^[20]^ As to our patient, the tumor originated from the diaphragm, the cells were abundant and dense under the microscope, the heteromorphic character was obvious, the tumor size was over 20 cm, some tumors showed obvious necrotic hemorrhage, CD34 is positive, especially the level of Ki67 was up to 10%, so it was in line with the diagnosis of malignant tumor.

Previously reported cases of MSFT of the diaphragm had extensive bases, and the diaphragm was reconstructed with artificial diaphragm patch because of the extensive resection. In contrast, the tumor in this case was extremely localized, with a distinct pedicle derived from the diaphragm, which could be easily removed en-block. Since the diaphragm could be sutured without tension, artificial diaphragm patch was not used. MSFT has a high recurrence rate and tumor-related mortality, but long-term survival of the patients could reach about 54% to 89% after en-block resection, but as to the palliative resection, the recurrence may occur within 24 months after initial treatment, once metastasis occurs, the prognosis is poor.^[21–24]^ There is no consensus on the safe distance of resection but rapid intraoperative frozen section pathological examination may be helpful for clinical management.

## 4 Conclusions

The present case indicated that MSFT should be considered in the diagnosis for giant tumors originating in the diaphragm, and immunohistochemical examination is essential for diagnosis. Even if the tumor is large, the presence of a pedunculated narrow base facilitates primary suturing of the diaphragm and eliminates the need for artificial reconstruction of diaphragm.

## Author contributions

**Conceptualization:** Dan Liu.

**Funding acquisition:** Wang Yun.

**Project administration:** Wang Yun.

**Resources:** Zheng Yu, Zhang Han-lu, Wang Zi-hao.

**Writing – review & editing:** Dan Liu.
